# Filamentous Fungal Infections in a Tertiary Care Setting: Epidemiology and Clinical Outcome

**DOI:** 10.3390/jof7010040

**Published:** 2021-01-09

**Authors:** Miriam Van den Nest, Gernot Wagner, Martin Riesenhuber, Constantin Dolle, Elisabeth Presterl, Gerald Gartlehner, Deddo Moertl, Birgit Willinger

**Affiliations:** 1 Division of Clinical Microbiology, Department of Laboratory Medicine, Medical University of Vienna, Waehringer Guertel 18-20, 1090 Vienna, Austria; miriam.vandennest@meduniwien.ac.at; 2 Department of Infection Control and Hospital Epidemiology, Medical University of Vienna, Waehringer Guertel 18-20, 1090 Vienna, Austria; elisabeth.presterl@meduniwien.ac.at; 3 Department for Evidence-Based Medicine and Evaluation, Danube University Krems, Dr.-Karl-Dorrek-Strasse 30, 3500 Krems, Austria; gernot.wagner@donau-uni.ac.at (G.W.); gerald.gartlehner@donau-uni.ac.at (G.G.); 4 Department of Medicine II, Division of Cardiology, Medical University of Vienna, Waehringer Guertel 18-20, 1090 Vienna, Austria; martin.riesenhuber@meduniwien.ac.at; 5 Clinical Institute for Physical Medicine and Rehabilitation, University Hospital St. Poelten, Karl Landsteiner University of Health Sciences, Dunant-Platz 1, 3100 St. Poelten, Austria; constantin.dolle@stpoelten.lknoe.at; 6 RTI International, 3040 East Cornwallis Road, P.O. Box 12194, Research Triangle Park, NC 27709-2194, USA; 7 Clinical Department of Internal Medicine III, University Hospital St. Poelten, Karl Landsteiner University of Health Sciences, Dunant-Platz 1, 3100 St. Poelten, Austria; deddo.moertl@stpoelten.lknoe.at

**Keywords:** filamentous fungi, invasive fungal infections, mold infections

## Abstract

Information on the distribution of filamentous fungal pathogens, which cause potential life-threatening invasive infections mostly in immunocompromised persons, is of great importance. The aim of this study was to evaluate the epidemiology and clinical outcome in patients with infections due to filamentous fungi at the University Hospital of Vienna, Austria. We conducted a retrospective observational study and consecutively included patients of any age with filamentous fungal infections between 2009 and 2017. The classification for probable and proven invasive filamentous fungal infections was based on the European Organization for Research and Treatment of Cancer/Invasive Fungal Infections Cooperative Group (EORTC) criteria or the expert opinion of an experienced clinical mycologist. We included 129 patients (median age: 52 years; 47.3% female) with episodes of 101 proven and probable invasive and 35 localized filamentous fungal infections (16 sinus, 14 eye, one ear, and four deep cutaneous). *Aspergillus fumigatus* alone accounted for 50.3% of the fungi, which was followed by the Mucorales group (13.7%) and *Fusarium* spp. (8.5%). Diagnosis was mainly based on culture findings. The lung was the most frequent site of infection. The 30-day and 90-day overall mortality of invasive fungal infections was 30.2% and 42.7%, respectively. We observed a high all-cause mortality among patients with invasive filamentous fungal infections. Prospective data collection in a nationwide registry would be necessary to provide important information on surveillance to clinicians and other decision-makers.

## 1. Introduction

Filamentous fungi are spreading ubiquitously in our environment, and invasive infections caused by those microorganisms are rising [1]. In particular, immunocompromised patients are at increased risk of developing an infection after exposure to filamentous fungi [1]. Patients with hematological diseases, after stem-cell or solid organ transplantation, and patients under immunosuppressive therapy in general are especially susceptible to fungal infections [2]. Further risk factors include chronic diseases such as chronic obstructive pulmonary disease (COPD), cystic fibrosis, and recent invasive procedures or trauma [1,2]. Thus, invasive fungal infections primarily affect patients in a poor state of health. Despite antifungal therapy, the mortality of invasive fungal infections remains high [3,4,5]. Klingspor et al. observed an overall 90-day mortality of 50.5% [3]. Immediate diagnosis and appropriate antifungal treatment is important to improve prognosis [6]. 

The therapy and diagnostic work-up of filamentous fungal infections is challenging since fungi are naturally found in the environment. This has several implications, including not only colonization of human surfaces and contamination of specimens with false-positive results but also delays in the diagnosis of filamentous fungal infections [6,7,8]. The European Organization for Research and Treatment of Cancer/Invasive Fungal Infections Cooperative Group (EORTC) and the National Institute of Allergy and Infectious Diseases Mycoses Study Group (MSG) have established guidelines to identify proven and probable invasive fungal infections, taking into account factors concerning microbiology as well as patient and clinical presentation [8].

Filamentous fungi such as *Aspergillus fumigatus* are among the most common pathogens causing opportunistic invasive fungal infections worldwide [9]. Nevertheless, over the last years, studies have reported a change in the spectrum of fungi causing invasive infections [2,10]. Understanding the national epidemiology and spectrum of fungal pathogens is of great importance to support clinicians, microbiologists, mycologists, and local health authorities. In Austria and other European countries, multi-center data on invasive filamentous fungal infections are scarce. The incidence rates for invasive aspergillosis are roughly estimated at 4.1 per 100,000 per year for Austria [11]. A publication of the Austrian Aspergillus Registry provided comprehensive prospective data on invasive mold infections in immunocompromised patients from the years 2007 and 2008, but those do not reflect the current epidemiological trends [12]. Recent data showing national trends in the spectrum of filamentous fungal pathogens causing invasive infections are currently lacking.

Therefore, the aim of this study was to evaluate the epidemiology and clinical outcome of filamentous fungal infections in a tertiary care center in Austria between 2009 and 2017.

## 2. Materials and Methods

### 2.1. Study Design

We conducted a single-center, retrospective observational study at the Division of Clinical Microbiology, Department of Laboratory Medicine, and the Department for Infection Control and Hospital Epidemiology, Medical University of Vienna. For this study, we obtained approval from the Ethics Committee of the Medical University of Vienna, Austria (EK Nr. 1006/2014).

### 2.2. Study Participants 

We consecutively included patients of any age admitted to one of the clinical departments of Vienna General Hospital, Medical University Vienna, with at least one probable or proven invasive filamentous fungal infections as well as localized filamentous fungal infections (sinus, eye, ear, and deep cutaneous) between January 2009 and August 2017. For probable or proven invasive infection, we did not consider cases with only one positive polymerase chain reaction (PCR) result, as they do not match the EORTC criteria [8]. We also excluded cases with suspected contamination due to isolation of a combination of fungal species not known for causing an infection in the respective organ and lack of clinical symptoms.

### 2.3. Study Setting

The Vienna General Hospital, Medical University of Vienna is a large tertiary care hospital, serving as reference center with large hemato-oncology, bone marrow, and solid organ transplantation units. Domestic and international patients are transferred to this center, as it offers highly specialized medical care and expertise.

### 2.4. Outcomes

The primary outcome of interest was the number and species of filamentous fungi causing probable and proven invasive filamentous fungal infections as well as localized filamentous fungal infections. The classification for probable and proven invasive filamentous fungal infections was based on the EORTC criteria [8] or the expert opinion of an experienced clinical mycologist. Positive culture findings from specimens obtained from sterile sites or positive histological findings were classified as a proven infection. For probable infections, the host factors, clinical features, physicians’ assigned diagnosis, and mycological evidence of infection were taken into account. Localized infections were defined by positive culture findings from the respective body sites and clinical features such as the corresponding symptoms and diagnosis assigned by clinician.

In cases where different fungi were isolated from the same specimen, we excluded any fungi suspicious for contamination; otherwise, we considered the infections polymicrobial. If multiple organs were affected, the infections were classified as disseminated if the same fungus species was obtained within three months; otherwise, we classified these infections as separate, new infections. Infections involving the bloodstream were also classified as disseminated infections. The secondary outcomes of interest were the affected organs, diagnostic methods, and all-cause mortality rates at 30 and 90 days.

### 2.5. Microbiological Diagnostics, Data Sources, and Variables

All specimens were collected and processed by the Division of Clinical Microbiology using standard microbiological methods throughout the study period. Samples were inoculated onto Sabouraud Dextrose agar, CHROMagarCandida^®^, Brain–Heart Infusion Agar slants, and Sabouraud-Glucose broth (all Becton Dickinson, Heidelberg, Germany) and incubated at both 35–37 °C and 28–30 °C up to three weeks. Fungal species were identified by colony morphology and microscopic features such as specific macro- and microconidia or sequence analysis. Upon request by clinicians, specimens were analyzed using in-house fungal broad-spectrum and/or *Aspergillus* reverse transcription polymerase chain reaction (RT-PCR), both targeting the internal transcribed spacer 2 (ITS2) region of the ribosomal DNA gene cluster to detect fungal DNA. Further, galactomannan assay from serum and bronchoalveolar lavage was performed on demand. The beta-D-Glucan and the lateral-flow device test for the detection of *Aspergillus* spp. were not performed at our institution during the study period. Whenever samples were sent to the pathology department, they were examined with standard histological methods, including periodic acid–Schiff (PAS) staining, if fungal infection was suspected.

We used electronic medical records and microbiology reports to collect demographic and microbiological data (age, gender, underlying risk factors and diseases, date of specimen collection, site of collection and infection, fungal specimen, method of identification). We used electronic medical records and cause-of-death statistics (Statistics Austria) to obtain information on patients’ vital status on 31 December 2017, and if deceased, the date and cause of death. We considered the date of specimen collection as the date of onset of infection.

### 2.6. Statistial Analyses

We performed descriptive statistical analyses and present the categorical variables as absolute frequencies and percentages. We summarized the continuous variables as a median and interquartile range. We calculated the time from the diagnosis of the first probable or proven infection (i.e., date of specimen collection) to the all-cause death or end of follow-up. Patients were censored if they were alive at the end of the follow-up period on 31 December 2017. We performed a Kaplan–Meier analysis and obtained estimates with 95% confidence intervals for the all-cause mortality at 30 and 90 days postdiagnosis. We conducted all statistical analyses with IBM SPSS Statistics software 26.0 (IBM Corp: Armonk, NY, USA) and Microsoft Excel (Microsoft Corporation, Redmond, WA, USA).

## 3. Results

### 3.1. Patient Characteristics

**Figure 1:**
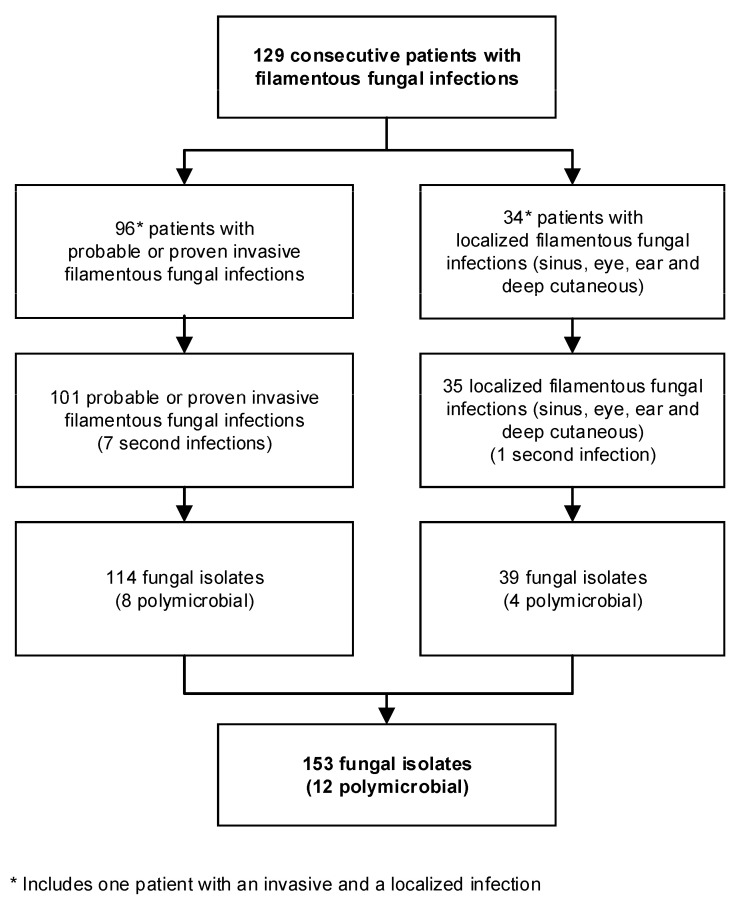
Flow chart shows number of patients, infections, and isolates included in analysis.

Overall, we analyzed the data of 129 patients with 101 invasive (64 proven, 37 probable) and 35 localized filamentous fungal infections (16 sinus, 14 eye, one ear, and four deep cutaneous) between 2009 and 2017. One patient had both an invasive and a localized infection. Figure 1 shows the number of patients with one or more infections and fungal isolates. 

The median age was 52 years (range 4–82 years); 61 (47.3%) patients were female. Table 1 shows the details of the patients’ characteristics for patients with probable or proven invasive infections and localized infections separately. The risk factors were equally distributed between female and male patients. The most common cause of acquired immunosuppression was immunosuppressive therapy, due to solid organ transplantation, hematologic malignancies, or other types of malignancies. Together, high-risk patients with hematologic malignancies or after solid organ transplantation accounted for 59.4% (57 of 96) of the patients with probable or proven invasive infections; 46.9% (45 of 96) of these patients had a history of solid organ transplantation. Among those, 35 had underwent lung transplantation. Among 34 patients with localized filamentous fungal infections, eleven patients (32.4%) suffered from immunosuppression. Half of the patients had none of the risk factors listed below, compared to 10.4% of patients with probable or proven invasive infection.

### 3.2. Number and Species of Isolated Filamentous Fungi

**Figure 2:**
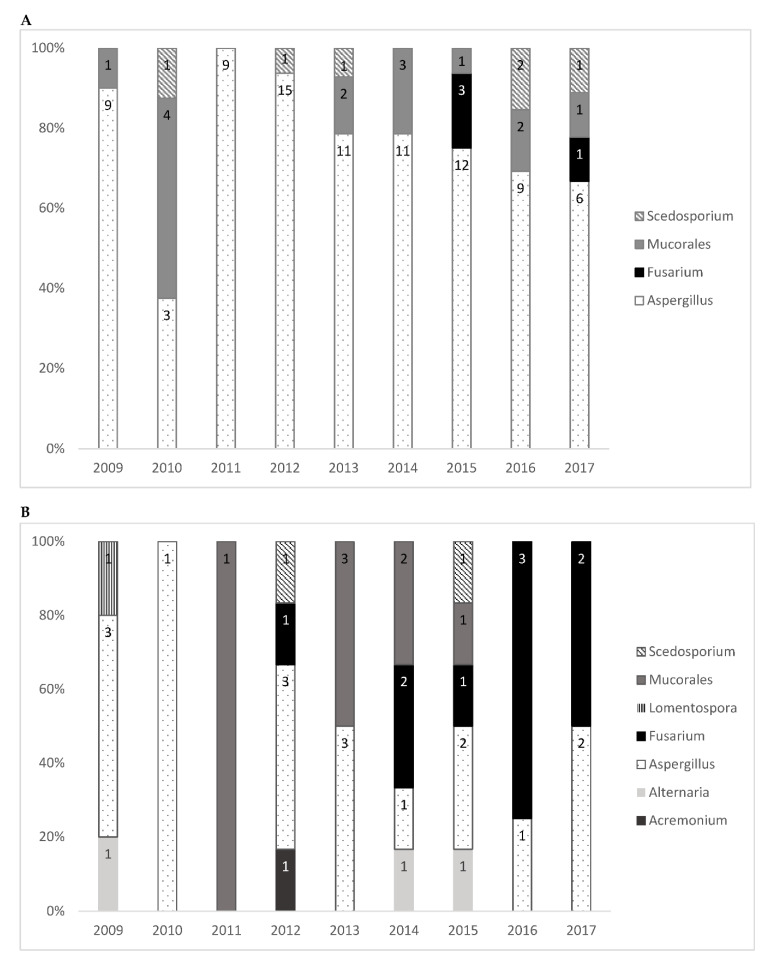
Filamentous fungal isolates by genera/order of fungi and year for (**A**) the four most common genera among probable or proven invasive infections and (**B**) for all genera of localized infections.

Overall, 153 fungi were identified: 68 (44.4%) in proven invasive, 46 (30.1%) in probable invasive, and 39 (25.5%) in localized infections. The species most frequently identified was *Aspergillus*, which accounted for 66.0% of the isolated fungi. *Aspergillus fumigatus* alone accounted for 50.3% of the fungi, which was followed by the Mucorales group (13.7%) and *Fusarium* spp. (8.5%). Table 2 shows the number of isolated filamentous fungi of each species; not all fungi could be identified at the species level. Table A1 and Table A2 of Appendix A provide the detailed distribution of fungal isolates by year. Figure 2 illustrates the number of filamentous fungal isolates according to the four most common genera during the study period for probable or proven invasive infections and for all genera among localized infections. *Fusarium* spp. infections were observed in the years 2012 to 2017 only.

Regarding high-risk patients with probable or proven invasive fungal infections, patients after lung transplantation were most frequently affected by *Aspergillus* spp. (85.7%; 30 of 35). In patients with probable or proven invasive infection and hematologic malignancies, 64.3% (9 of 14) of the isolates were *Aspergillus* spp. and 28.6% (4 of 14) of the isolates identified were Mucorales. Table 3 shows number of fungal isolates of the four most common genera among high-risk patients. *Scedosporium apiospermum* caused infections in three patients after lung transplantation, including one patient with cystic fibrosis as an underlying disease. The disseminated infection in this patient affected lung, soft tissue, and heart and thus was not considered a colonization. Of the eight polymicrobial infections, five (62.5%) contained more than one *Aspergillus* spp., and seven (87.5%) affected the lung; one infection caused by *Rhizopus microsporus* and *Aspergillus fumigatus* affected the lung, stomach, and heart of a cancer patient. 

For rare fungi, we identified one case of histoplasmosis in a patient after kidney transplantation; upon diagnosis, the patient was admitted to an intensive care unit (ICU). The patient died one day after *Histoplasma capsulatum* was identified in a pulmonal and a pericardial specimen. *Paecilomyces variotii* was found in the bloodstream of a patient with severe heart disease suffering from ischemic cardiomyopathy and chronical renal failure three days after implantation of left ventricular assist device.

### 3.3. Organs Affected by Filamentous Fungi

The majority of probable or proven invasive infections included pulmonary infection (91 of 101; 90.1%), followed by disseminated infections (18 of 101; 17.8%). In 15 of 101 (14.9%) probable or proven invasive infections, more than one organ was affected; only two of them did not involve the lung. Of the patients suffering from a disseminated fungal infection, 52.9% (nine of 17) were admitted to the ICU. Among those, six were post-transplant and three were hematologic patients. Of the disseminated infections, 64.7% (11 of 17) involved *Aspergillus* spp. Organs affected by disseminated infections included the lung, heart, brain, and soft tissue. In three patients, fungi were detected in a total of three organs. 

Table 4 shows the spectrum of filamentous fungi detected in each organ. *Aspergillus* spp. was the most frequently identified in every organ, except for in soft tissue and wound infections, where Mucorales were more frequent (five of 12; 41.7%), and eye infections were most often caused by *Fusarium* spp. (eight of 14; 57.1%). 

### 3.4. Diagnostic Methods

The diagnosis of filamentous fungal infections was primarily based on culture findings (Table 5). For 97 of 153 (63.4%) fungal isolates, the diagnosis was only based on a positive culture. Among those, 40 (26.1%) were also identified by histopathologic investigation. For 20 (13.1%) fungal isolates, PCR was the method of identification, which was accompanied by either histology or culture. 

For 64 proven invasive infections, 82.4% (56 of 68) of fungi were detected by culture, 26.5% (18 of 68) were detected additionally by histopathology, 12 were detected by additional positive PCR, and 51.5% (35 of 68) were diagnosed by culture alone. Regarding 37 probable invasive infections, 44 fungi were identified in the bronchoalveolar lavage fluid using culture methods. For four fungi, additional histopathologic results indicating, but not proving, fungal infection were found. Overall, seven patients had a positive test result for galactomannan.

### 3.5. Mortality among Patients with Probable or Proven Invasive Filamentous Fungal Infection

During the observation period (median 48 months; range: 5–105 months), 58 of 96 (60.4%) patients with a probable or proven invasive filamentous fungal infection died. Among patients with pulmonary infections, 49 of 86 (57.0%) died as well as all patients with disseminated infections. Table 6 shows the number of events and all-cause mortality at 30 and 90 days for all patients with invasive filamentous fungal infections and the two most frequent genera separately.

Of the patients with disseminated infections, 52.9% were in the ICU when diagnosed and had a high 90-day mortality (13 of 17; 76.5%). Within 90 days after diagnosis, four of 35 patients with localized infections died. 

## 4. Discussion

This retrospective observational study describes the epidemiology of filamentous fungal infections in a tertiary care center during a period of nine years. Diagnosis was mainly based on culture-based methods. Over this period, *Aspergillus* spp. was the most commonly isolated genus. *Aspergillus fumigatus* alone accounted for more than half of the cases, followed by Mucorales and *Fusarium* spp. We observed no fungal infections caused by *Fusarium* spp. between 2009 and 2011, but there were 13 infections from 2012 to 2017. The organ most frequently affected in all patient groups was the lung. We observed a low rate of polymicrobial infections. In our study population, 30% of the patients died within one month, which probably reflects the critical health condition of those patients. However, we were not able to assess whether the cause of death was related to fungal infections and how risk factors and underlying conditions influenced mortality.

In our study, the most frequent underlying condition was solid organ transplantation, in contrast to the findings from another study [3]. This may be due the fact that our tertiary care center is specialized in solid organ transplantations. Most of these patients underwent lung transplantation, and, accordingly, the lung was the organ affected by invasive fungal infections in the majority of those patients. In contrast to other studies [3,13], hematological patients accounted for only 14% of our study population. Our population further involved many so-called new susceptible patients, such as COPD or diabetes mellitus patients.

Diagnosis was mainly based on culture-based methods, although PCR techniques gained importance over time. A great number of pulmonary infections could only be classified as probable invasive infections, reflecting the difficulties in interpreting the specimens collected from the respiratory tract. Although histopathology was a part of diagnostics in almost one-third of all infections, it primarily supported findings from other methods in proving the infection. 

In general, our findings are in accordance with those of other observational studies with the majority of patients suffering from aspergillosis [3,4,5]. Aspergillus, in particular *A. fumigatus*, is known to be a common cause of invasive fungal infections in immunocompromised patients, especially when neutropenic or on corticosteroids [14]. Patients with lung diseases or after lung transplantation are also affected by invasive aspergillosis [14]. In our study, 81% of invasive filamentous fungal infections among lung transplant and 90% among COPD patients were caused by *Aspergillus* spp. In general, after the inhalation of *Aspergillus* spores, manifestation of the infection can take up to several months, which causes a delay in diagnosis and treatment. Obtainment of sterile material poses another difficulty in the accurate diagnosis of invasive aspergillosis, which is reflected in the proportion of probable invasive infections among patients with pulmonary aspergillosis.

In contrast to other observational studies, we did not observe an increase in infections with Mucorales over time. Generally, Mucorales cause opportunistic infections in patients with immunosuppression or metabolic dysfunction [15]. Mucorales often affect the sinuses, lung, skin, soft tissue and, more rarely, the gastrointestinal tract or central nervous system. We identified one case of gastric mucormycosis in a patient with gastric cancer. Unlike other studies [15], we did not find a high rate of diabetes mellitus patients among the mucormycosis cases. This may be due to an underdiagnosis of mucormycosis, as diagnosis is challenging, and culture lacks sensitivity [15]. Most of the patients with probable or proven mucormycosis suffered from a hematologic malignancy or had received a solid organ transplantation; thus, mortality was high, and 10 of 11 patients died within 90 days after diagnosis. In patients with previous lung transplantations, Mucorales only accounted for a small proportion of the isolates identified, whereas in patients with hematologic malignancies, more than two-thirds suffered from mucormycosis. For Mucorales, not only is diagnosis challenging, but treatment is as well, as there are no breakpoints for antifungal susceptibility testing, and options for antifungal therapy are limited [2]. Therefore, knowledge of local epidemiology is crucial to warrant adequate antifungal treatment. 

From year 2012 onwards, only, *Fusarium* spp. were found, which was possibly due to emerging resistances and the increased usage of antifungal prophylaxis in hematopoietic stem cell and solid organ transplantation patients, or due to rising awareness among ophthalmologists. Most *Fusarium* species invariably show a very high resistance to various antifungal agents and could therefore be missed in an antifungal prophylactic regimen. *Fusarium* spp. lead to local infections in the skin and eyes; for invasive infections, mortality rates above 75% are reported [2]. In our cohort, *Fusarium* spp. mostly caused eye infections, primarily in non-high-risk patients. It is well known that *Fusarium* eye infections are more common in tropical and subtropical countries. However, with the increasing use of soft contact lenses, fungal keratitis has also become a problem in urban areas with moderate climates. The German National Reference Center for Mycology observed a case series of 22 eye infections caused by Fusarium species, with species of the *Fusarium solani* species complex being the dominating etiological agent [16]. In contrast to these observations, we mainly found that species of the *Fusarium oxysporum* species complex caused eye infections, although we must admit that the number of fungal eye infection cases is too small to draw any conclusions concerning the epidemiology of fungal eye infections.

This study has several limitations. First, we retrospectively collected the data on invasive fungal infections based on electronic medical records. Since different sources of bias are inherent to this study design, we might have missed or misclassified relevant information. Second, we did not collect information on antifungal prophylaxis and therapy as well as complete comedication. Third, our single-center findings from a large tertiary care university hospital providing care for patients requiring specific treatment are not transferable to other settings. Finally, we were not able to calculate the prevalence and incidence of filamentous fungal infections in patients with certain underlying conditions and risk factors. 

However, the findings of our study provide thorough insights into the spectrum of fungal infections in patients admitted to a tertiary care hospital and might trigger future research in this important clinical field. A nationwide registry with a prospective collection of data from hospitalized patients with invasive fungal infections would provide continuous infections surveillance with important epidemiological information for decision-makers.

## 5. Conclusions

In patients with various underlying acute or chronic conditions, *A. fumigatus* was the most common cause of invasive filamentous fungal infections. The all-cause mortality was high among patients with invasive filamentous fungal infections. Most frequently, we found infections of the lung. Prospective data collection in a nationwide registry should be promoted as it would provide important information on the epidemiology and outcome.

## Figures and Tables

**Table 1:** Characteristics, risk factors, and underlying conditions upon diagnosis for patients with probable or proven invasive infection and localized infections (*n* = 129). One patient had both an invasive and a localized infection.

	Female	Male	Total
**Patients with probable or proven invasive infections**	***n* = 44**	***n* = 52**	***n* = 96**
Age (years), median (IQR)	50.0 (36.0–60.0)	58.0 (34.5–65.8)	52.5 (35.3–64)
Age, group, *n* (%)			
0–20	3 (6.8%)	6 (11.5%)	9 (9.4%)
21–45	13 (29.5%)	12 (23.0%)	25 (26.0%)
46–65	21 (47.7%)	21 (40.4%)	42 (43.8%)
66-85	7 (15.9%)	13 (25.0%)	20 (20.8%)
Medical history, *n* (%) ^1^			
Solid organ transplantation	19 (33.2%)	26 (50%)	45 (46.9%)
Hematologic malignancies ^2^	6 (13.6%)	6 (11.5%)	12 (12.5%)
Allogeneic stem cell transplantation	1 (2.3%)	4 (7.7%)	5 (5.2%)
Solid cancer ^3^	3 (3.1%)	8 (8.3%)	11 (11.5%)
Chemotherapy or radiotherapy ^4^	7 (15.9%)	8 (15.4%)	15 (15.6%)
Diabetes	7 (15.9%)	9 (17.3%)	16 (16.7%)
Cystic fibrosis	4 (9.1%)	1 (1.9%)	5 (5.2%)
COPD	6 (13.6%)	15 (28.8%)	21 (15.5%)
Heart disease	16 (36.4%)	10 (19.2%)	26 (21.9%)
HIV infection	-	-	0 (0.0%)
Congenital or acquired immunosuppression	23 (52.3%)	29 (55.8%)	52 (54.2%)
Surgery within 30 days prior to diagnosis of fungal infection	9 (20.5%)	12 (23.1%)	21 (21.9%)
**Patients with localized infections**	***n* = 18**	***n* = 16**	***n* = 34**
Age (years), median (IQR)	45.5 (30.3–67.5)	59.0 (42.8–64.8)	53.5 (33.8–65.5)
Age, group, *n* (%)			
0–20	3 (16.7%)	1 (6.3%)	4 (11.8%)
21–45	6 (33.3%)	4 (25.0%)	10 (29.4%)
46–65	4 (41.0%)	8 (50.0%)	12 (35.3%)
66–85	5 (27.8%)	3 (18.8%)	8 (23.5%)
Medical history, *n* (%) ^1^			
Solid organ transplantation	2 (11.1%)	3 (18.8%)	5 (14.7%)
Hematologic malignancies ^5^	3 (16.7%)	3 (18.8%)	6 (17.6%)
Allogeneic stem cell transplantation	1 (5.6%)	-	1 (2.9%)
Solid cancer ^6^	1 (5.6%)	2 (12.5%)	3 (8.8%)
Chemotherapy or radiotherapy ^4^	4 (41.0%)	2 (12.5%)	6 (17.6%)
Diabetes	-	2 (12.5%)	2 (5.9%)
Cystic fibrosis	-	1 (6.3%)	1 (2.9%)
COPD	-	-	0 (0.0%)
Heart disease	2 (11.1%)	1 (6.3%)	3 (8.8%)
HIV infection	-	-	0 (0.0%)
Congenital or acquired immunosuppression	6 (33.3%)	5 (31.3%)	11 (32.4%)
Surgery within 30 days prior to diagnosis of fungal infection	1 (5.6%)	5 (31.3%)	6 (17.6%)

Abbreviations: IQR, interquartile range; COPD, chronic obstructive pulmonary disease; HIV, human immunodeficiency virus; n, number of patients. ^1^ Patients could have more than one diagnosis. ^2^ Includes patients with acute and chronic myeloid leukemia, acute and chronic lymphoblastic leukemia, and Non-Hodgkin lymphoma. ^3^ Includes patients with mamma, cervical, stomach, prostate and lung cancer, seminoma, neuroblastoma, meningeoma, spindle cell tumor, brainstem ganglioma. ^4^ Includes patients with solid or hematologic malignancies. ^5^ Includes patients with acute myeloid leukemia, chronic lymphoblastic leukemia, and Non-Hodgkin lymphoma. ^6^ Includes patients with lung and stomach cancer, neuroblastoma.

**Table 2:** Number and species of isolated filamentous fungi

Detected Fungus	Proven and Probable Invasive	Localized Infections (Sinus, Eye, Ear, and Deep Cutaneous)	Total
***Acremonium* spp. ^1^**	-	**1 (2.6%)**	**1 (0.7%)**
***Alternaria* spp.**	**1 (0.9%)**	**3 (7.7%)**	**4 (2.6%)**
*Alternaria alternata*	-	1 (2.6%)	1 (0.7%)
*Alternaria infectoria*	-	1 (2.6%)	1 (0.7%)
Other ^1^	1 (0.9%)	1 (2.6%)	2 (1.3%)
***Aspergillus* spp.**	**85 (74.6%)**	**16 (41.0%)**	**101 (66.0%)**
*Aspergillus flavus*	6 (5.3%)	3 (7.7%)	9 (5.9%)
*Aspergillus fumigatus*	66 (57.9%)	11 (28.2%)	77 (50.3%)
*Aspergillus nidulans*	2 (1.8%)	1 (2.6%)	3 (2.0%)
*Aspergillus niger*	8 (7.0%)	-	8 (5.2%)
*Aspergillus terreus*	3 (2.6%)	1 (2.6%)	4 (2.6%)
***Fusarium* spp.**	**4 (3.5%)**	**9 (23.1%)**	**13 (8.5%)**
*Fusarium oxysporum* species complex	1 (0.9%)	3 (7.7%)	4 (2.6%)
*Fusarium fujikuroi* species complex *(F. proliferatum)*	1 (0.9%)	1 (2.6%)	2 (1.3%)
*Fusarium solani* species complex	1 (0.9%)	2 (5.1%)	3 (2.0%)
Other ^1^	1 (0.9%)	3 (7.7%)	4 (2.6%)
***Histoplasma* spp.**	1 (0.9%)	**-**	**1 (0.7%)**
*Histoplasma capsulatum*	1 (0.9%)	-	1 (0.7%)
***Lomentospora* spp.**	**-**	**1 (2.6%)**	**1 (0.7%)**
***Lomentospora prolificans***	-	**1 (2.6%)**	**1 (0.7%)**
**Mucorales**	**14 (12.3%)**	**7 (17.9%)**	**21 (13.7%)**
*Lichtheimia corymbifera*	1 (0.9%)	3 (7.7%)	4 (2.6%)
*Lichtheimia ramosa*	1 (0.9%)	-	1 (0.7%)
*Mucor circinelloides*	-	1 (2.6%)	1 (0.7%)
*Rhizomucor pusillus*	1 (0.9%)	1 (2.6%)	2 (1.3%)
*Rhizopus arrhizus*	6 (5.3%)	1 (2.6%)	8 (5.2%)
*Rhizopus microsporus*	4 (3.5%)	-	4 (2.6%)
*Rhizopus* spp. ^1^	1 (0.9%)	-	1 (0.7%)
***Paecilomyces* spp.**	**1 (0.9%)**	**-**	**1 (0.7%)**
*Paecilomyces variotii*	1 (0.9%)	-	1 (0.7%)
***Scedosporium* spp.**	**6 (5.3%)**	**3 (7.7%)**	**8 (5.2%)**
*Scedosporium apiospermum*	6 (5.3%)	2 (5.1%)	8 (5.2%)
***Schizophyllum* spp.**	**1 (0.9%)**	**-**	**1 (0.7%)**
*Schizophyllum commune*	1 (0.9%)	-	1 (0.7%)
***Trichoderma* spp. ^1^**	**1 (0.9%)**	**-**	**1 (0.7%)**
Total	114	39	153

Abbreviations: spp., species ^1^ Species not further identified.

**Table 3:** Filamentous fungal isolates in probable or proven invasive infection of the four most common genera among high-risk patients.

Genus/Order of Fungi	Hematologic Malignancies ^1^	Allogeneic Stem Cell Transplantation	Lung Transplantation	Heart Transplantation	Combined Heart and Lung Transplantation	Kidney Transplantation
*Aspergillus* spp.	9	4	34	1	3	4
*Fusarium* spp.	-	1	2	-	-	-
Mucorales	4	1	3	2	1	1
*Scedosporium* spp.	-	-	3	-	-	-
Total	13	6	42	3	4	5

Abbreviations: spp., species. ^1^ Includes patients with acute and chronic myeloid leukemia, acute and chronic lymphoblastic leukemia and Non-Hodgkin lymphoma.

**Table 4:** Fungal spectrum according to organ.

Genus/Order of Fungi	Proven and Probable Invasive Infections								Localized Infections		
	Lung	Disseminated	CNS	Heart	Soft Tissue and Wounds ^1^	Stomach ^1^	Sinus ^1^	Eye ^1^	Sinus and Ear	Eye	Deep Cutaneous
*Alternaria* spp.	-	-	-	-	1	-	-	-	-	1	2
*Aspergillus* spp.	82	11	3	2	1	1	1	1	13	3	-
*Fusarium* spp.	3	1	-	-	-	-	-	-	-	8	1
*Histoplasma* spp.	1	1	-	1	-	-	-	-	-	-	-
*Lomentospora* spp.	-	-	-	-	-	-	-	-	-	1	-
Mucorales	11	4	1	2	4	1	-	-	6	-	1
*Paecilomyces* spp.	-	1	-	-	-	-	-	-	-	-	-
*Scedosporium* spp.	5	1	-	1	2	-	-	-	1	1	-
*Schizophyllum* spp.	1	-	-	-	-	-	-	-	-	-	-
*Trichoderma* spp.	1	-	-	-	-	-	-	-	-	-	-
Total	104	19	4	6	8	2	1	1	20	14	4

Abbreviations: CNS, central nervous system; spp., species. ^1^ Organs affected as part of disseminated infection.

**Table 5:** Diagnostics methods by genera for probable or proven invasive and localized infections.

Genus/Order of Fungi	Number of Fungal Isolates	Culture	PCR	Histology	Galactomannan Assay
**Probable or proven invasive infections**					
*Alternaria* spp.	1 (0.9%)	1	1	1	-
*Aspergillus* spp.	85 (74.6%)	75	10	22	7
*Fusarium* spp.	4 (3.5%)	4	-	-	-
*Histoplasma* spp.	1 (0.9%)	1	-	1	-
Mucorales	14 (12.3%)	11	3	7	-
*Paecilomyces* spp.	1 (0.9%)	1	-	-	-
*Scedosporium* spp.	6 (5.3%)	6	-	1	-
*Schizophyllum* spp.	1 (0.9%)	1	-	-	-
*Trichoderma* spp.	1 (0.9%)	1	-	-	-
**Localized infections**					
*Acremonium* spp.	1 (2.6%)	1	-	-	-
*Alternaria* spp.	3 (7.7%)	3	-	-	-
*Aspergillus* spp.	16 (41.0%)	16	1	4	-
*Fusarium* spp.	9 (23.1%)	8	3	1	-
*Lomentospora* spp.	1 (2.6%)	1	-	1	-
Mucorales	7 (17.9%)	7	1	4	-
*Scedosporium* spp.	2 (5.1%)	2	-	1	-

Abbreviations: PCR, polymerase chain reaction; spp., species.

**Table 6:** Kaplan–Meier estimates of all-cause mortality for patients with invasive filamentous fungal infections at different time points.

		30 Days		90 Days	
	*n*	*n Died*	% (95% CI)	*n Died*	% (95% CI)
Invasive fungal infection	96	29	30.2% (22.1–40.5)	41	42.7% (33.5–53.2)
*Aspergillus* spp. ^1^	76	20	26.3% (17.9–37.8)	30	39.5% (29.5–51.4)
Mucorales ^1^	11	9	81.8% (55.8–97.2)	10	90.9% (66.7–99.5)

Abbreviations: CI, confidence interval; n, number of patients; spp., species. ^1^ Includes patients with polymicrobial infections.

## Data Availability

Data not available on request due to restriction.

## Appendix A

**Table A1:** Spectrum of isolated filamentous fungi in probable or proven infections per year.

	2009	2010	2011	2012	2013	2014	2015	2016	2017	Total
***Alternaria* spp. ^1^**	**-**	**1**	**-**	**-**	**-**	**-**	**-**	**-**	**-**	**1 (0.9%)**
***Aspergillus* spp.**	**9**	**3**	**9**	**15**	**11**	**11**	**12**	**9**	**6**	**85 (74.6%)**
*Aspergillus flavus*	1	-	-	2	2	1	-	-	-	6 (5.3%)
*Aspergillus fumigatus*	7	3	7	10	8	8	10	7	6	66 (57.9%)
*Aspergillus nidulans*	-	-	-	1	-	1	-	-	-	2 (1.8%)
*Aspergillus niger*	1	-	-	1	1	1	2	2	-	8 (7.0%)
*Aspergillus terreus*	-	-	2	1	-	-	-	-	-	3 (2.6%)
***Fusarium* spp.**	**-**	**-**	**-**	**-**	**-**	**-**	**3**	**1**	**1**	**4 (3.5%)**
*Fusarium oxysporum* species complex	-	-	-	-	-	-	1	-	-	1 (0.9%)
*Fusarium fujikuroi* species complex *(F. proliferatum)*	-	-	-	-	-	-	1	-	-	1 (0.9%)
*Fusarium solani* species complex	-	-	-	-	-	-	1	-	-	1 (0.9%)
Other ^1^	-	-	-	-	-	-	-	-	1	1 (0.9%)
***Histoplasma* spp.**	**-**	**-**	**-**	**-**	**1**	**-**	**-**	**-**	**-**	**1 (0.9%)**
*Histoplasma capsulatum*	-	-	-	-	1	-	-	-	-	1 (0.9%)
**Mucorales**	**1**	**4**	**-**	**-**	**2**	**3**	**1**	**2**	**1**	**14 (13.2%)**
*Lichtheimia corymbifera*	-	-	-	-	-	1	-	-	-	1 (0.9%)
*Lichtheimia ramosa*	-	-	-	-	1	-	-	-	-	1 (0.9%)
*Rhizomucor pusillus*	-	-	-	-	-	-	-	1	-	1 (0.9%)
*Rhizopus arrhizus*	-	3	-	-	-	2	-	-	-	6 (5.3%)
*Rhizopus microsporus*	1	-	-	-	1	-	1	-	1	4 (3.5%)
*Rhizopus* spp. ^1^	-	1	-	-	-	-	-	-	-	1 (0.9%)
***Paecilomyces* spp.**	**-**	**-**	**-**	**1**	**-**	**-**	**-**	**-**	**-**	**1 (0.9%)**
*Paecilomyces variotii*	-	-	-	1	-	-	-	-	-	1 (0.9%)
***Scedosporium* spp.**	**-**	**1**	**-**	**1**	**1**	**-**	**-**	**2**	**1**	**6 (5.3%)**
*Scedosporium apiospermum*	-	1	-	1	1	-	-	2	1	6 (5.3%)
***Schizophyllum* spp.**	**-**	**-**	**-**	**-**	**1**	**-**	**-**	**-**	**-**	**1 (0.9%)**
*Schizophyllum commune*	-	-	-	-	1	-	-	-	-	1 (0.9%)
***Trichoderma* spp. ^1^**	**1**	**-**	**-**	**-**	**-**	**-**	**-**	**-**	**-**	**1 (0.9%)**
Total	11 (9.6%)	9 (7.9%)	9 (7.9%)	17 (14.9%)	16 (14.0%)	14 (12.3%)	16 (14.0%)	13 (11.9%)	9 (7.9%)	114

Abbreviations: spp., subspecies. ^1^ Species not further identified.

**Table A2:** Spectrum of isolated filamentous fungi auricular, ocular, deep cutaneous, and sinus localized infections per year.

	2009	2010	2011	2012	2013	2014	2015	2016	2017	Total
***Acremonium* spp. ^1^**	**-**	**-**	**-**	**1**	**-**	**-**	**-**	**-**	**-**	**1 (2.6%)**
***Alternaria* spp. ^1^**	**1**	**-**	**-**	**-**	**-**	**1**	**1**	**-**	**-**	**3 (7.7%)**
*Alternaria alternata*	1	-	-	-	-	-	-	-	-	1 (2.6%)
*Alternaria infectoria*	-	-	-	-	-	-	1	-	-	1 (2.6%)
Other ^1^	-	-	-	-	-	1	-	-	-	1 (2.6%)
***Aspergillus* spp.**	**3**	**1**	**-**	**3**	**3**	**1**	**2**	**1**	**2**	**16 (41.0%)**
*Aspergillus flavus*	-	1	-	-	-	-	1	-	1	3 (7.7%)
*Aspergillus fumigatus*	3	-	-	2	2	1	1	1	1	11 (28.2%)
*Aspergillus nidulans*	-	-	-	-	1	-	-	-	-	1 (2.6%)
*Aspergillus terreus*	-	-	-	1	-	-	-	-	-	1 (2.6%)
***Fusarium* spp.**	**-**	**-**	**-**	**1**	**-**	**2**	**1**	**3**	**2**	**9 (23.1%)**
*Fusarium oxysporum* species complex	-	-	-	1	-	1	-	1	-	3 (7.7%)
*Fusarium fujikuroi* species complex *(F. proliferatum)*	-	-	-	-	-	-	-	-	1	1 (2.6%)
*Fusarium solani* species complex	-	-	-	-	-	-	1	1	-	2 (5.1%)
Other ^1^	-	-	-	-	-	1	-	1	1	3 (7.7%)
***Lomentospora* spp.**	1	-	-	-	-	-	-	-	-	1 (2.6%)
*Lomentospora prolificans*	1	-	-	-	-	-	-	-	-	1 (2.6%)
**Mucorales**	**-**	**-**	**1**	**-**	**3**	**2**	**1**	**-**	**-**	**6 (15.4%)**
*Lichtheimia corymbifera*	-	-	1	-	2	-	-	-	-	3 (7.7%)
*Mucor circinelloides*	-	-	-	-	1	-	-	-	-	1 (2.6%)
*Rhizomucor pusillus*	-	-	-	-	-	1	-	-	-	1 (2.6%)
*Rhizopus arrhizus*	-	-	-	-	-	1	1	-	-	2 (5.1%)
***Scedosporium* spp.**	**1**	**-**	**-**	**1**	**-**	**-**	**1**	**-**	**-**	**2 (5.1%)**
*Scedosporium apiospermum*	-	-	-	1	-	-	1	-	-	2 (5.1%)
Total	5 (11.4%)	1 (2.6%)	1 (2.6%)	6 (15.4%)	6 (15.4%)	6 (15.4%)	6 (15.4%)	4 (10.3%)	4 (10.3%)	39

Abbreviations: spp., subspecies. ^1^ Species not further identified.
